# Invited Discussion

**DOI:** 10.1007/s11695-024-07344-9

**Published:** 2024-06-25

**Authors:** David B. Sarwer

**Affiliations:** Temple University College of Public Health, Philadelphia, PA USA

**Keywords:** Metabolic and bariatiatric surgery, Utilization, Social support, Advocacy

Many researchers and clinicians in the field of metabolic and bariatric surgery (MBS) also have spent a great deal of professional, if not personal time, advocating for greater utilization of the surgical treatment of obesity. This is particularly true in countries like the United States where public and private insurance companies have long resisted greater access to care. The earliest concerns raised by those third-party payers were related to the efficacy and safety of MBS. The world-wide body of evidence has largely demonstrated that the most widely used procedures are efficacious and safe, particularly when performed by well-trained, experienced multidisciplinary teams.

Concerns about the durability of weight loss and improvements in comorbidities also have been raised. Again, the research community answered those issues. Next were questions about the return on investment of the initial costs of MBS as compared to health care savings over time. While a growing number of studies have documented the health care savings that are realized from MBS, it is important to remember that there are very few other examples in health care where such concerns have been used as a barrier to a potentially life-saving, and certainly life-enhancing, procedure like MBS. This issue, in particular, has raised important questions about the role that weight bias, stigma, and discrimination plays in decision-making related to MBS.

Unfortunately, MBS remains profoundly underutilized, with estimates suggesting that approximately 1% of individuals who meet the weight and comorbidity requirements undergoing surgery each year. Utilization also is impacted by race and ethnicity. In the United States, for example, rates of clinically severe obesity are highest among persons of color. Yet, they represent approximately one-quarter of Americans who undergo surgery each year.

Other logistical issues impede the path to the operating room. In many programs in the United States, it takes approximately 6 months from a patient’s initial visit with the treatment team to the day of surgery. During that time, patients are required to complete a number of preoperative evaluations, including with a mental health professional. Many are mandated to participate in a medical weight management program which is implemented, in theory, to promote weight loss in advance of surgery. For all of these reasons, and others, anecdotal reports suggest that only a small percentage of patients who complete that initial appointment with the team end up having surgery with the program.

The study by Kapera and colleagues looks at the issue of utilization from a novel perspective—social support. Several members of the multidisciplinary team ask candidates for surgery about issues of social support during the preoperative evaluation process. This is to both help understand how family members and friends may have contributed to the development of obesity but, more importantly, to help patients identify individuals who can provide support both before and after surgery. The large sample of patients enrolled in the study completed a validated, patient-reported outcome measure which assessed the quality of relationships in the family. While 35% of the sample underwent MBS, greater cohesion and emotional expressiveness in the family were associated with a greater likelihood of undergoing surgery. This pattern of results did not vary by race or ethnicity.

The results remind us of the important role that family support can play in MBS. While this support may positively impact utilization, it likely plays an important role in postoperative outcomes as well. We know that 10–20% of patients experience suboptimal weight losses or early and significant weight regain. We also know that similar percentages of patients struggle with a range of significant mental health issues—disordered eating, depression, substance misuse, and suicidality—during the first postoperative decade. Positive social support may play a role in reducing the occurrence of these untoward outcomes.

As we continue to advocate for greater utilization of MBS around the world, it also is important to retain focus on the important role that family, friends, and members of the multidisciplinary team can play in identifying problematic behavioral issues postoperatively and directing patients to additional care to support lifelong success.

